# Occurrence of Pesticides, Mycotoxins, and Heavy Metals in Distilled Alcoholic Beverages: A Review of Contaminants and Health Risks

**DOI:** 10.3390/foods14081303

**Published:** 2025-04-09

**Authors:** Tomislav Rot, Sunčana Gavran, Jurislav Babić, Ante Lončarić

**Affiliations:** 1 Faculty of Food Technology Osijek, Josip Juraj Strossmayer University of Osijek, Franje Kuhača 18, 31000 Osijek, Croatia; tomislav.rot@inspecto.hr (T.R.); jurislav.babic@ptfos.hr (J.B.); 2 Faculty of Tourism and Rural Development in Požega, Josip Juraj Strossmayer University of Osijek, Vukovarska ul. 17, 34000 Požega, Croatia

**Keywords:** distilled spirits, pesticides, mycotoxins, heavy metals, migration

## Abstract

Distilled alcoholic beverages may contain pesticides, mycotoxins, and heavy metals originating from raw materials, environmental factors and technological processes. This review paper analyses the existing regulations related to these contaminants, their impact on health and the risk assessment associated with their consumption. Sources of contamination are discussed, including the influence of climatic conditions and emerging environmental risks on the occurrence of contaminants in raw materials, as well as the transfer of all contaminants during the distillation process. Furthermore, analytical detection methods and strategic measures to reduce consumer exposure are reviewed. The results of the review highlight the need for continued research, improvement of control methods and adaptation of regulatory standards in line with new scientific knowledge.

## 1. Introduction

Spirits are alcoholic beverages intended for human consumption, which have specific sensory properties and contain an alcohol content of at least 15% by volume. They are produced by direct distillation of fermented agricultural raw materials, maceration of herbs in ethyl alcohol of agricultural origin, or by adding flavorings, sweeteners and other food products. They can also be produced by mixing strong alcoholic beverages with other alcoholic components [1]. Distilled alcoholic beverages or spirits contain the greatest concentration of alcohol. By distilling ethanol obtained from fermenting agricultural products, such as fruits, grains, or vegetables, they remove almost all of their naturally occurring compounds, except water and ethyl alcohol [2]. Generally, spirits processing involves the preparation of raw materials, fermentation, distillation, and aging [3]. At the concentrations usually found in alcoholic drinks, hundreds of chemicals serve as flavoring agents, but there may also be many toxic and carcinogenic compounds present [4]. Some of the most famous spirits are whiskey, brandy, rum, vodka, gin, and tequila. All these drinks are characterized by a unique production technology, and they are produced from different raw materials such as grains (whiskey, vodka, gin), fruit (brandy, rum, gin, tequila), vegetables (vodka, gin) and sugar (vodka, rum, gin) [5]. Fruit spirits are also very popular; they are produced by fermenting various fruits, vegetables, and even grains, and finally distilling the fermented mash [6]. Alcoholic spirits, like food in general, are susceptible to contamination by various contaminants, some of which are shown in Table 1.

Mycotoxins are secondary metabolites produced by a wide variety of filamentous fungi, most important of them *Aspergillus*, *Fusarium*, *Penicillium*, and *Alternatia* [7,8]. Mycotoxins are known to cause a variety of toxic effects in humans and animals, including hepatotoxicity, nephrotoxicity, neurotoxicity, mutagenicity, carcinogenicity, and immunosuppression [9]. When exposed to mycotoxins, people can develop numerous diseases such as allergies, reduced immune response, and even cancer [10].

Apart from this, other contaminants are found in the environment or on process equipment, such as pesticides and heavy metals [3,11]. Heavy metals are essential micronutrients and play a role in cellular biochemistry and physiological functions, but they also pose a number of health risks [12]. Pesticides are becoming more prevalent in agriculture due to increasing food demands [2]. Some of the reasons why pesticides are used in the cultivation of raw materials intended for the production of distilled alcohol are to combat diseases and insects, increase crop yields, improve their quality, and extend their shelf life. Whether pesticides appear in the distilled alcoholic beverage depends on the physical and chemical properties of the pesticides during the production process [13,14]. Exposure to pesticides and heavy metals can have an immunotoxic and carcinogenic effect on the body. It can also cause oxidative stress, changing immune and hormonal level reactions in the human body, which can lead to the formation of tumors [15]. The following are comprehensive reviews of the recent literature on the presence of these contaminants in distilled alcoholic spirits, their regulation, migration pathways, and potential health risks.

## 2. Regulation and Legislation

Given their high popularity, frequency of consumption, and various contaminants in raw materials, spirits are a potential risk to human health. In order to ensure the quality and safety of spirits, many countries and international organizations have introduced standards and regulations related to alcoholic beverages.

Regulation (EU) 2019/787 [1] of the European Parliament and Council regulates the production, labeling, and protection of spirit drinks within the European Union. It lays down definitions, criteria for raw materials and production processes, rules on geographical indications, and conditions for placing these products on the market.

The European Union’s Regulation (EC) No 396/2005 [16] regulates the maximum residue levels (MRLs) for pesticides in food, including raw materials used in the production of alcoholic beverages. Pesticide residues in raw materials must not exceed permissible limits in order to maintain the safety of the final product. In addition, the Commission Regulation (EU) 2023/915 [17] of 25 April 2023 has defined MLs for certain contaminants such as mycotoxins and heavy metals in food and raw materials, and one of them is patulin (PAT), the maximum value of which is determined specifically for spirit alcohols.

In the USA, the presence of pesticides, mycotoxins, and heavy metals in food is regulated by the US Food and Drug Administration for the sake of consumer health.

## 3. Climate Change and Emerging Environmental Risks

Food systems are being threatened by climate change, affecting food security as well as food safety and quality [18]. In addition to affecting responses to abiotic stress, immune impulses and pathogen survival, environmental conditions can also affect pathogen development and survival [19].

Climate change plays a very important role in the appearance of mycotoxins in raw materials for strong alcoholic beverages. Species and concentrations of certain mycotoxins are found to be in a correlation with meteorological conditions in the year of crop harvest, thus some species prefer hot and drought seasons, while others prefer warm weather and moisture [20,21]. Temperature, humidity, and precipitation changes can favor the growth of fungi such as *Aspergillus*, *Penicillium*, and *Fusarium*, which are known mycotoxin producers. These fungal genera produce the greatest number of mycotoxins, of which the most important ones are aflatoxins (AFT), fumonisins (FUM), ochratoxins, trichothecenes, and zearalenone (ZEA). A combination of high temperatures, droughts, and sudden increases in humidity is most conducive to the development of these fungi, thereby increasing the danger of contamination of raw materials like grains and grapes [22,23]. In Southern and Central Europe, climatic conditions that favor the growth of *Aspergillus flavus* will likely increase the risk of AFT contamination in maize in the next 30 years [24]. Magan and Aldred [25] emphasize that aflatoxin B1 (AFB1), produced by *A. flavus*, is spreading geographically due to global warming, and previously unaffected regions are being contaminated. This contamination is particularly prevalent in maize, a major ingredient in whiskey and other spirits production. As a result of the extremely dry weather in those years, *A. flavus* has become one of the most important pathogens in maize. In the case of a temperature increase of 2 °C, the risk of AFT will increase in some areas in Mediterranean Europe [26]. The most important factor besides temperature is the levels of CO_2_. CO_2_ concentrations in the atmosphere can increase the aggressiveness of *Fusarium* species, resulting in the production of FUM and deoxynivalenol (DON). Due to the widespread distribution of these mycotoxins on wheat and barley, whiskey and beer production are prime targets [24].

**Table 1:** Summary table of analyses of individual contaminants.

Contaminant Type	Examples		Origin	Influencing Factors	Literature
Pesticides	herbicides fungicides insecticides acaricide plant growth regulators		grain raw materials residues fruit raw materials residues	pesticide stability distillation efficiency	[13,27]
Mycotoxins	AFT DON FUM ZEA T-2/HT-2 OTA PAT		fungal contamination of raw materials	climate conditions storage conditions	[28,29]
Heavy Metals	Essential	Cu Zn Co Cr Mn Fe	environmental pollution equipment contamination	water source soil contamination distillation setup	[4]
	Non-essential	Pb Cd Hg As			

As well as affecting mycotoxins, climate change can also affect raw material microbiomes. Microorganisms that act as natural antagonists against mycotoxigenic fungi can be reduced by an increase in temperature. As a result, harmful species are allowed to grow uncontrolled, therefore increasing contamination risk [23]. The impact of climate change could be mitigated by adopting integrated agricultural management systems, such as crop rotation, proper irrigation methods and the use of biological plant protection agents. Mycotoxins can also be digitally monitored and forecasted so that producers can make timely decisions [24].

Climate change, especially rising temperatures and altered precipitation patterns, is expected to impact crop yields and pesticide use. Due to changes in pest prevalence and pesticide efficiency, pesticide application is expected to increase in quantity, frequency, and variety. It is also possible that climate change will accelerate the degradation of pesticides through volatilization, moisture, and sunlight exposure, potentially reducing the concentrations of pesticides in the environment [30]. According to this, climate change will certainly affect the presence of pesticides in the environment in the future. Higher temperatures may accelerate their degradation, reducing exposure, while heavy rainfall after application may increase concentrations in water. Pesticides such as azoxystrobin, difenoconazole, and MCPA pose significant environmental risks, highlighting the need of reducing their use [31].

There are several reasons why heavy metals appear in food, such as their natural existence in the Earth’s crust at different concentrations or their gradual deposition in the environment by humans [32]. Food contamination with heavy metals is also a result of the depletion of deposits, direct air pollution, irrigation of soil with polluted water and groundwater pollution [33]. These metals travel through the atmosphere and water via dispersion and deposition, accumulating in soils and water sediments. These sediments act as secondary sources, releasing heavy metals back into the environment. Climate conditions play a crucial role in this process. Rising temperatures can reduce soil water content while increasing evapotranspiration, which in turn causes more resuspension of soil dust particles containing heavy metals [34]. As persistent environmental pollutants, heavy metals accumulate and biomagnify within biological systems, causing severe health and ecological harm [35].

## 4. Occurrence of Mycotoxins in Cereal-Based Raw Materials

Currently, the most used raw materials for the production of distilled alcoholic beverages are, among others, cereals. The most common examples of these cereals are rye, wheat, and maize [36]. Since they are exposed to different weather conditions and climate changes in the field, as well as poor handling and storage conditions, grains are contaminated with various types of toxigenic fungi and with them, their secondary metabolites, mycotoxins [37]. Among hundreds of known mycotoxins, the focus of most research and regulations are on AFT and ochratoxin A (OTA), *Fusarium* mycotoxins FUM, ZEA, DON, and T-2/HT-2 toxins, since they contaminate numerous foods and occur worldwide [9].

The main producers of AFT are members of the *Aspergillus* species. They contaminate different cereal grains in the field such as wheat, barley, corn, rice, and sorghum, mostly in hot and humid conditions. Of the aflatoxins listed, according to the International Agency for Research on Cancer (IARC), aflatoxin B1 is classified as a Category 1 human carcinogen [38].

The following group of mycotoxins mainly produced by *Fusarium verticillioides* (syn. *Fusarium moniliforme*) and *Fusarium proliferatum* fungi in the field are fumonisins, which contaminate various types of crops such as corn, wheat, sorghum, and barley [39,40]. More than 30 FUM homologs have been identified so far, and the most widespread and toxic member is fumonisin B1 (FB1), which is classified as a possibly carcinogenic to humans by IARC in Group 2B [38].

The next family of mycotoxins, ochratoxins, are produced mainly by *Aspergillus ochraceus*, *Aspergillus carbonarius*, and *Penicillium verrucosum* in different matrices such as dry beans, corn, peanuts, rapeseeds, etc., [41]. Ochratoxins can be divided into three different groups (A, B, and C) based on differences in characteristic functional groups. According to Al-Anati and Petzinger [42], OTA is the most toxic member of the ochratoxin family, and in addition to the acute immunotoxicity and hepatotoxicity it can cause, animal studies have shown that it can induce immunotoxicity and act as a carcinogen [40,43].

Another well-known mycotoxin that is formed in the field and during postharvest storage is DON, which belongs to the β-trichothecene group. It is produced by *Fusarium* fungi, specifically *Fusarium culmorum* and *Fusarium graminearum*, which are the most important causes of fusarium head blight, a cereal disease that attacks wheat, barley, triticale, and oats [44]. Although it is less toxic than T-2 and HT-2 toxins, which belong to type A trichothecenes, it contaminates cereals and cereal products in a high proportion [45,46]. According to the impact on human health by IARC, DON is classified in Group 3, not classifiable as to its carcinogenicity to humans [38].

The mycotoxin ZEA is primarily produced by various *Fusarium* species, such as *Fusarium culmorum*, *Fusarium graminearum*, and *Fusarium sporotrichioides*, and occurs in temperate regions on various types of cereals such as barley, oats, sorghum, wheat, and rice [47]. In addition to contamination in the field, which is the most common, contamination of grains can occur due to storage with high humidity levels [48]. Similar to DON, ZEA is classified as a Group 3 carcinogen by IARC [38].

PAT is a mycotoxin produced by the genera *Aspergillus*, *Penicilium*, and *Byssochlamys* through secondary metabolism. At high concentrations, it exhibits immunotoxic properties but is not carcinogenic to humans [49]. Unlike the previously mentioned mycotoxins, which are characteristic of cereals, PAT can be detected as a natural contaminant mainly in pome fruits, such as apples, but also in other fruits, such as hawthorn [50]. In addition, it often appears in fruit juices, especially apple juices produced from unqualified fruit [3].

## 5. Occurrence of Pesticides in Fruit-Based Raw Materials

Due to their unique aroma, distilled fruit drinks are very popular. This type of drink is produced from various fruits, both cultivated and wild, and the most common representatives are plums, apples, cherries, pears, apricots, and quinces [51].

Since pesticides are commonly used in the production of cultivated fruits, among the most common commodities containing pesticide residues are apples, oranges, grapefruit, apricots, peaches, etc. [52]. According to another study, the fruits with the highest levels of pesticide residues were apples, papayas, strawberries, pears, grapes, and citrus fruits. In addition to their role in production and protection, pesticides are associated with a wide range of hazards to human health, ranging from short-term effects such as headaches and nausea, to chronic effects such as cancer, reproductive problems, and endocrine disruption [53].

In general, the term pesticides covers a wide range of substances including insecticides, fungicides, herbicides, rodenticides, molluscicides, nematicides, plant growth regulators, and others [54]. According to their chemical structure, pesticides can be divided into the following groups: organochlorines, organophosphates, carbamic, and thiocarbamic derivates, carboxylic acids and their derivates, urea derivates, heterocyclic compounds, phenol and nitrophenol derivates, hydrocarbons, ketones, aldehydes, fluorine-containing compounds, copper-containing compounds, metal–organic and inorganic compounds, natural, and synthetic pyrethroids [55].

Since there are different fruits that are treated with a large number of different pesticides, the rest of this chapter will present research on pesticide residues found in one type of fruit, apples. According to research published in their paper by Kowalska et al. [56], the apple harvests from 2012 and 2020 were compared, according to which in 2012, as many as 26 different pesticide residues were quantified in the observed apples, and in 2020, only 7 of them were quantified. As listed, the following pesticides were detected in 2012: boscalid (fungicide), carbendazim (fungicide), chlorpyrifos (acaricide, insecticide), bupirimate (fungicide), difenoconazole (fungicide), diphenylamine (plant growth regulator), disulfoton (insecticide), hexythiazox (acaricide, insecticide), fenazaquin (acaricide), malathion (acaricide, insecticide), propargite (acaricide), pyraclostrobin (fungicide, plant growth regulator), pyrimethanil (fungicide), thiophanate methyl (fungicide), thiacloprid (insecticide), triflumuron (insecticide), flusilazole (fungicide), pirimicarb (insecticide), trifloxystrobin (fungicide), methoxyfenozide (insecticide), thiodicarb (insecticide), epoxiconazole (fungicide), hexaflumuron (insecticide), triadimenol (fungicide), indoxacarb (insecticide), and cyprodinil (fungicide). Much fewer identified pesticide residues in apples were found during the 2020 harvest, namely: boscalid (fungicide), pyraclostrobin (fungicide, plant growth regulator), captan (fungicide), fludioxonil (fungicide), tetrahydrophthalimide (THPI) (metabolite of captan–fungicyd), fluopyram (fungicide), tebuconazole (fungicide).

## 6. Metals in Alcoholic Beverages and Public Health Implications

Heavy metals are a group of compounds with high atomic numbers and a density five times greater than the density of water. They are naturally occurring, mostly found in the Earth’s crust, and cannot be easily degraded, so they persist for a long period [57]. Essential heavy metals are naturally present in the human body and are beneficial to human health. This group consists of metals such as copper, zinc, cobalt, chromium, manganese, and iron, which are needed by every living organism for various biochemical and physiological functions [58]. However, these metals can be toxic if the concentration is higher than the maximum permissible limit for the organism [59], like copper and zinc, which are considered essential for a living organism, but excessive intake can lead to disorders and diseases [60]. The toxic properties of copper are manifested in the bioaccumulative properties of the Cu^2+^ ion, which can travel through the food chain. When the intake of Cu^2+^ ions is increased, various diseases can occur, such as heart disease, brain damage, circulatory system damage, gastrointestinal irritation, and necrotic changes in the liver and kidneys [61].

Non-essential heavy metals, on the other hand, can be toxic to body cells even at low concentrations, and they have no biological role in the living organisms [58]. Among the most important representatives of this group of metals are certainly lead (Pb), cadmium (Cd), mercury (Hg), and arsenic (As), which pose a threat to human health if exposure occurs. Long-term exposure to these elements could lead to progressive progression of physical diseases as well as neurological degenerative conditions [57]. It is known that exposure to Cd and Hg leads to various diseases that are mostly manifested in reduced kidney function and reproductive capacity, hypertension, tumors, and liver dysfunction, while exposure to lead leads to kidney failure, liver damage, hearing impairment, intellectual disability [62], damages the nervous system, and causes brain disorders [3]. In addition to its harmful and toxic effects, Pb is considered a major environmental health risk [60]. Moreover, some of the metals are classified as carcinogens (Cr, As, Cd), while some are possible carcinogens (Ni, Co, Pb) [63].

In most alcoholic beverages, the main ingredients and raw materials are mostly plant materials, in which metal bioaccumulation occurs mainly through uptake from the soil. The reason for metal accumulation in the soil is mainly the use of fertilizers, pesticides, contaminated surface water, sewage sludge, and wastewater [58]. In addition, the contamination of cereals with heavy metals also occurs due to the rapid industrialization and urbanization of agricultural areas [21]. It is possible to find them in raw materials for the production of fruit spirits [64], and possible sources are equipment used in all parts of the production process like distillation, aging, bottling, and storage. Accordingly, numerous metalloids and metals such as As, Cd, Cr, Co, Cu, Fe, Mn, Ni, Sn, Pb, and Zn were found in them [4]. They may be frequently present at levels of public health concern, particularly in off-label spirits, but are rarely found at levels that can cause acute toxicity [64]. However, if levels of heavy metal ions are exceeded, they can be harmful to human health due to their toxic properties [65]. The presence of metals in food and beverages should be monitored frequently to ensure that they are safe for human consumption [66].

## 7. Migration into Distillates

The residual amount of pesticides, excluding those lost during fermentation and distillation, in distilled spirits can be found in stills if some of the pesticides migrate to the stills [13]. While distillation may reduce the concentration of pesticide residues, it is not completely effective [67]. According to a study conducted by [27], analyzing the migration of 249 pesticides from the raw material to the distillate, it was found that 220 pesticides were not detected in the distillate. Therefore, it can be concluded that the distillation process reduces the risk of exposure to pesticides. Nevertheless, several pesticides (cycloate, cadusafos, diallate, ethoprophos, and thiometon), which may pose a risk, have significant transfer ratios (Table 2). In another study by [13], five pesticides (terbufos, fenthion, iprobenfos, flutolanil, and ethoprophos) contaminated the raw material with about 5.1 to 70 times the MRL, and the distillation migration was from 0 to 14.3%, which led to the conclusion that some pesticides migrate into the distillate in single-stage distillation. However, in the same study, the fermented liquefaction was contaminated with six pesticides (fenthion, terbufos, ethoprophos, iprobenfos, oxadiazon, and flutolanil), and after distillation with a distillation column, none of the pesticides were detected in the distilled spirit [13].

**Figure 1:**
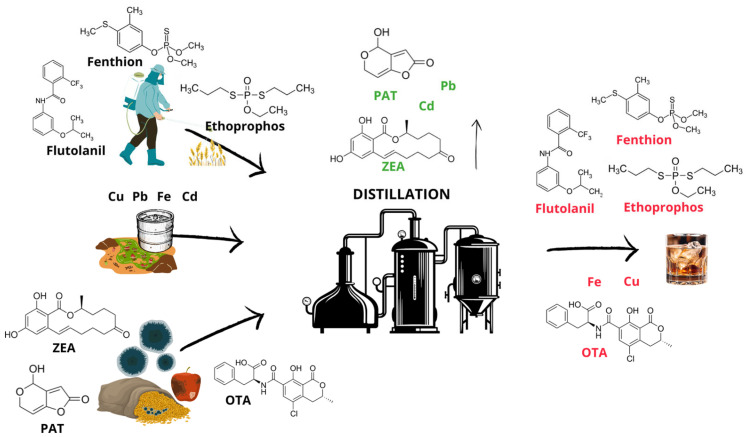
Schematic representation of contaminant migration.

Contamination with mycotoxins can be found in a variety of grains, fruits, and other raw materials used in making whiskey, brandy, and vodka. Although distillation greatly reduces the quantity of mycotoxins, these toxic substances are still detectable in the final product, according to certain studies [28]. Since mycotoxins remain persistent in raw materials, any poor processing or lack of appropriate monitoring could easily result in their migration into distilled beverages [29]. Due to the high temperatures generated during distillation, mycotoxins are thermally decomposed and separated from the volatile alcohol fractions, thereby reducing their concentration. OTA can be degraded only to a lesser extent by heat and only at temperatures of 180 °C and above, and testing the thermal stability of aflatoxin in its pure form, it was found that a heating temperature of approximately 150 °C almost completely degrades AFB1 [68]. Shin and Lee [28] investigated the migration of selected mycotoxins during the process of distillation and their presence in distillates prepared by pilot-scale continuous distillation. This research has proven that, even in cases of contamination of raw materials with certain levels of the tested mycotoxins, their migration into the distillate is negligible when a continuous distillation column is used. Thus, only 0.19 µg/L of OTA was detected in the distillate (Table 2) with an alcohol content of 94–95% vol, and the migration took place in three consecutive distillations (0.11–0.22 µg/L). These results indicate a low risk of mycotoxin presence in the final product when applying a continuous distillation process. Pietri et al. [69] have shown that mycotoxins survive fermentation and distillation, although their concentrations decrease dramatically. As alcohol is steam distilled, fermented, and distilled, its raw ingredients are removed. The presence of migrating mycotoxins within distillate products can indeed be mitigated by a combination of prevention measures that include appropriate raw material selection, efficient fermentation conditions, enhanced distillation processes and vigilant observation [70]. While some studies suggest that barrel aging may reduce mycotoxin levels due to adsorption to the barrel wall, others suggest that contamination may occur due to barrel fungal spores. In order to reduce the risk of mycotoxins, care must be taken in the selection of materials and their storage [71]. Figure 1 illustrates the pathways of migration of mycotoxins, pesticides, and heavy metals into distilled spirits.

Various sources, including raw materials, brewing processes, equipment, bottling, aging, and storage contribute to the migration of metals into alcoholic beverages [72]. According to available research, the presence of metalloids and metals such as As, Cd, Cr, Cu, Fe, Mn, Ni, Sn, Pb, and Zn has been determined in distilled spirits. The analysis of commercially available spirits shown in Table 2 (palinka, whiskey, vodka, brandy, rum, gin, tequila, absinthe) reveals variability in heavy metal concentrations with Cu ranging from 0.0 to 15.3 mg/L and Ni up to 77.16 mg/L. In unrecorded palinka, significantly higher concentrations of Cu up to 51.6 mg/L and Zn up to 16.96 mg/L were recorded, indicating potential issues in the distillation process [4]. Since fertilizers, fungicides, pesticides, and mineral fertilizers affect the metal content of raw materials, they can affect the Cd, Cu, Mn, Pb, and Zn content of the final product. Studies have shown that Mn and Cu can come largely from the oak barrels in which the product is aged, as well as the oak chips used in aging [73].

## 8. Risk Assessment

Considering the different production methods and product properties, according to the Codex Alimentarius, alcoholic beverages can be classified into beer and malt beverages, ciders and perry, grape wines (still, sparkling, fortified, mead) and distilled spirituous beverages containing more than 15% alcohol [74]. Globally, about 50% of total alcohol consumption is consumed in the form of spirits. This shows that the total consumption of pure alcohol in the form of spirits is equal to the total consumption of other alcoholic beverages, and this is due to the great popularity of spirits and the high proportion of alcohol that is concentrated during the distillation process [3].

A key measure for risk management is certainly the establishment of MLs for chemical contaminants in food, where it is necessary to take into consideration the amount, toxicity, and concentration of contaminants in the consumed food. The mere nonconformity of the product does not mean, according to the principles of risk assessment, that the product is toxic, but in order to ensure effective protection of public health, it is necessary to prevent such products from entering the market or being used as ingredients in other food [3].

It is extremely important to identify potential hazards and emerging ones in all stages of the product, from production to consumption. Taking the example of pesticides, during the rapid growth of food production in the last few decades, they began to be used to control insects, weeds and fungi, and several pesticides are known to be harmful to both the environment and human health. It is very difficult to control pesticide residues for all input raw materials in the production of distilled spirits, so manufacturers periodically inspect randomly selected raw materials, some of which are imported from countries with low levels of control and safety standards [13]. Although numerous countries and organizations have established standards for pesticide residues in various foods, such as wheat, corn, barley, and grapes, which are regularly used in the production of spirits, only a few studies have conducted a risk assessment for pesticides in spirits (Table 2). At the end of the research conducted by Shin et al. [13], it was concluded that the risk of detecting pesticides in distilled spirits can be reduced with proper distillation.

When it comes to the problem of contamination with mycotoxins, cereals stored for a long period of time pose a great risk. Therefore, it is very important to ensure the safety and quality of the product, regardless of whether mycotoxins transfer from the contaminated raw material to the distilled spirit or not. According to the research conducted by Shin and Lee [28], it was concluded that the mycotoxins AFB1, DON, and ZEA were not detected in the distillate after single-stage distillation (Table 2), which means that regardless of the amount of contamination of the raw material with these mycotoxins, after fermentation and distillation, they will not remain in the distillate. Regarding the impact on raw material contamination, *Lactobacillus* and *S. cerevisiae* showed the ability to reduce the concentrations of AFB1, DON, FUM, T-2, and ZEA, with the most significant detoxification occurring within 6 h, and stabilization followed after 24 h. These strains of microorganisms show potential for use as food and feed additives to mitigate mycotoxin contamination [75]. Some of the novel strategies to protect against mycotoxins include the development of genetically modified crops resistant to mycotoxigenic fungi and innovative detoxification techniques such as enzymatic treatments and adsorption agents. Mycotoxins can be reduced in the final products by using these methods, thereby reducing the risk to public health [76]. In order to avoid contamination with mycotoxin, distilled beverage producers must implement stringent quality control practices. Maintaining product safety requires the implementation of HACCP plans that specifically address mycotoxin monitoring, testing, and compliance with regulatory guidelines. In order to avoid the migration of mycotoxins into final products, the industry may benefit further from advances in predictive modeling and risk assessment tools [77].

The presence of heavy metals from raw materials in spirits can be prevented by the distillation process. For example, Pb in spirits mostly migrates from equipment and containers during the production process, distribution, and storage [3,58].

Quality assurance in the field of food contaminant detection is evolving over time, so it is essential that in the coming years the focus is on further improving the sensitivity, selectivity, and speed of analytical methods. Various innovations such as portable and miniaturized analytical devices have enabled the analysis of contaminants at the point of origin, whether in the field, factories, or markets. Regardless of the previously mentioned innovations, high-performance liquid chromatography combined with mass spectrometry (Table 3) will continue to be indispensable for the detection and quantification of known and unknown contaminants such as pesticides and mycotoxins at the trace level [78]. The most common techniques (Table 3) for testing trace metals in spirits are Electrothermal and Flame Spectroscorpy (ETAAS, FAAS), Inductively Coupled Plasma Atomic Emission Spectroscopy and Mass Spectrometry (ICP-AES, ICP-MS), and electrochemical methods, but some publications include TXRF spectrometry for the determination of metals in distilled alcoholic beverages [63].

In order to ensure the change and establishment of quality standards based on scientific claims, it is important that the risk assessment is based on a comprehensive detection method, toxicological information, and technological measures, and most importantly, it must be applicable in risk management. Therefore, in order to support the making of important decisions in risk management, risk assessment must consolidate and evaluate the available scientific evidence in an objective, transparent, and appropriate way [81]. To establish standards in the spirit drinks production technology, it is recommended that risk assessment be combined with toxicological approaches, whereby it is necessary to expand the comprehensive scope of contaminant analyzes in order to avoid potential risks as successfully as possible. Also, it is important that quality control measures for incoming raw materials, fermentation, distillation, storage, and equipment are implemented effectively and used appropriately, as this is the only way to ensure the reduction in most contaminants [3]. According to these premises, the determination of certain contaminants in frequently consumed spirits is of great importance for the nutritional, toxicological and food safety fields [60].

## 9. Conclusions

The presence of pesticides, mycotoxins, and heavy metals in distilled alcoholic spirits remains a significant concern, especially in the context of climate change, environmental conditions, and production practices. While industrially produced distillates generally contain low levels of contamination, recent findings reviewed in this paper confirm the migration of certain pesticides, ochratoxin A, metalloids, and heavy metals during the distillation process, highlighting the need for continued vigilance. Particular attention should be paid to traditional and unregistered products, which may pose a higher risk due to inadequate equipment and insufficient regulatory oversight.

To ensure consumer safety and public health, future efforts should focus on strengthening regulations, improving quality control of raw materials, and optimizing production processes to minimize contamination risks. Advances in analytical techniques will be crucial in detecting contaminant residues with greater accuracy. In addition, further research is needed to understand the mechanisms of contaminant migration during distillation and to develop effective mitigation strategies. A multidisciplinary approach involving food safety, analytical chemistry, and regulatory enforcement will be essential to preserving the quality and safety of distilled alcoholic beverages.

## Figures and Tables

**Table 2:** Contamination and migration of pesticides, mycotoxins, and heavy metals in distilled spirits.

Type of Spirit Beverage	Raw Material	Analyte	Spike (mg/L)	Conc. in Distillate (mg/L)	Literature
Sake	Fermented rice bran powder	Terbufos	0.02	ND ^2^	[13]
			0.04	0.0018	
		Iprobenfon	0.28	ND	
		Fenthion	0.14	0.0024	
			0.33	0.01	
		Flutolanil	1.27	0.01	
		Ethoprophos	0.35	0.05	
		Pb	10	ND	
		Cd	10	ND	
		AFB1	0.04	ND	[28]
		OTA	0.02	0.00019	
		DON	4	ND	
		ZEA	0.8	ND	
Barley shochu	Fermented barley mash	PAT	0.05	ND	[29]
		NIV	0.05	ND	
		DON	0.05	ND	
		AFG2	0.01	ND	
		AFG1	0.01	ND	
		AFB2	0.01	ND	
		AFB1	0.01	ND	
		HT-2	0.05	ND	
		T-2	0.05	ND	
		ZEA	0.05	ND	
		FB1	0.05	ND	
		OTA	0.05	ND	
		FB2	0.05	ND	
		Cycloate	0.05	0.022 ^1^	[27]
		Cadusafos	0.05	0.009 ^1^	
		Diallate	0.05	0.015 ^1^	
		Ethoprophos	0.05	0.015 ^1^	
		Thiometon	0.05	0.012 ^1^	
		Terbufos	0.05	0.008 ^1^	
Recorded spirits (n = 97): Palinka (n = 25), whiskey (n = 21), vodka (n = 16), brandy (n = 18), rum (n = 6), artificially flavored spirits (n = 5), gin (n = 3), tequila (n = 2), absinthe (n = 1)	-	Copper	-	0.0–15.3	[4]
		Cobalt	-	0.0–0.31	
		Chromium	-	0.0–0.71	
		Iron	-	0.0–34.63	
		Manganese	-	0.0–3.38	
		Nickel	-	0.0–77.16	
		Zinc	-	0.0–7.49	
		Tin	-	0.0–3.47	
Unrecorded spirits (n = 100): Palinka	-	Copper	-	0.0–51.60	
		Iron	-	0.0–11.91	
		Manganese	-	0.0–0.79	
		Nickel	-	0.0–6.86	
		Zinc	-	0.0–16.96	
		Tin	-	0.0–4.10	

^1^ Calculated according to the formula (transfer ratio (%) = (each pesticide’s conc. (µg/L) × 90 mL) × 100/(50 µg/L × 200 mL)). ^2^ Not detected.

**Table 3:** Comparison of instrumental techniques for the analysis of investigated contaminants.

Contaminants	Analysis Methods	Advantages	Disadvantages	Literature
Heavy metals	ICP/MS, ICP/OES	High sensitivity, ability to analyze multiple elements simultaneously	High operating costs, high argon consumption, complex sample prep, time-consuming	[65,79]
	TXRF	Fast, low cost, minimal sample preparation, for high-concentration samples	High detection limits, spectral interferences, less precise for trace elements	
	GFAAS	Improved sensitivity, lower detection limits	Complex sample preparation, slower analysis	
	FAAS	Fast analysis, relatively low cost	Lower sensitivity compared to other techniques	
Pesticides	GC/MS, GC/MS/MS	High sensitivity, good separation	Requires extensive sample preparation, expensive equipment	
	HPLC/MS, HPLC/MS/MS	Excellent selectivity, suitable for complex matrices	High operational costs, potential matrix interferences	
Mycotoxins	HPLC-FLD/UV/MS	High sensitivity, allows simultaneous detection of multiple mycotoxins, applicable for various matrices	Requires derivatization for some mycotoxins, expensive equipment, complex sample preparation	[80]
	LC-MS/MS	High selectivity and sensitivity, reliable identification, applicable to complex matrices	High operational costs, requires highly trained personnel	
	ELISA	Fast and simple screening method, enables high-throughput testing, requires low sample volume and minimal clean-up	Matrix interferences may affect accuracy, requires thorough validation for different food matrices	
	LFIA	Simple, fast results, low cost, suitable for large-scale on-site screening, no need for sample clean-up	Potential interferences, limited sensitivity for trace analytes	
	Biosensors	High sensitivity, real-time detection, possibility of multi-toxin analysis, portable, suitable for on-site testing	Matrix interference, antibody cross-reactivity, necessity of matrices’ validation	

## Data Availability

No new data were created or analyzed in this study. Data sharing is not applicable to this article.

## Abbreviations

The following abbreviations are used in this manuscript:

15-ADON	15-acetyl-deoxynivalenol
3-ADON	3-acetyl-deoxynivalenol
AFB1	aflatoxin B1
AFB2	aflatoxin B2
AFG1	aflatoxin G1
AFG2	aflatoxin G2
AFT	aflatoxins
As	arsenic
Cd	cadmium
Co	cobalt
Cr	chromium
Cu	copper
D3G	deoxinyvalenol-3-glucoside
DON	deoxynivalenol
FB1	fumonisin B1
Fe	iron
FUM	fumonisins
Hg	mercury
IARC	International Agency for Research on Cancer
MCPA	4-chloro-2-methylphenoxyacetic acid
ML	Maximum Level
Mn	manganese
MRL	Maximum Residue Level
Ni	nickel
NIV	nivalenol
OTA	ochratoxin A
PAT	patulin
Pb	lead
Rf	retention factor
SDHI	succinate dehydrogenase inhibitors
Sn	tin
THPI	tetrahydrophthalimide
TLC	thin-layer chromatography
ZEA	zearalenone
Zn	zinc
